# Induced cell fate transitions at multiple cell layers configure haustorium development in parasitic plants

**DOI:** 10.1242/dev.164848

**Published:** 2018-07-23

**Authors:** Takanori Wakatake, Satoko Yoshida, Ken Shirasu

**Affiliations:** 1 Graduate School of Science, The University of Tokyo, Bunkyo, Tokyo 113-0033, Japan; 2 RIKEN Center for Sustainable Resource Science, Yokohama, Kanagawa 230-0045, Japan; 3 Institute for Research Initiatives, Division for Research Strategy, Nara Institute of Science and Technology, Ikoma, Nara 630-0192, Japan; 4 Graduate School of Biological Science, Nara Institute of Science and Technology, 8916-5 Takayama-cho, Ikoma, Nara 630-0192, Japan

**Keywords:** Parasitic plant, Haustorium formation, Organogenesis, Cell fate transition, WOX4, HB15, CASP1, CESA7, Cellular reprogramming

## Abstract

The haustorium in parasitic plants is an organ specialized for invasion and nutrient uptake from host plant tissues. Despite its importance, the developmental processes of haustoria are mostly unknown. To understand the dynamics of cell fate change and cellular lineage during haustorium development, we performed live imaging-based marker expression analysis and cell-lineage tracing during haustorium formation in the model facultative root parasite *Phtheirospermum japonicum*. Our live-imaging analysis revealed that haustorium formation was associated with induction of simultaneous cell division in multiple cellular layers, such as epidermis, cortex and endodermis. In addition, we found that procambium-like cells, monitored by cell type-specific markers, emerged within the central region of the haustorium before xylem connection to the host plant. Our clonal analysis of cell lineages showed that cells in multiple cellular layers differentiated into procambium-like cells, whereas epidermal cells eventually transitioned into specialized cells interfacing with the host plant. Thus, our data provide a cell fate transition map during *de novo* haustorium organogenesis in parasitic plants.

## Abstract (precis)

**Highlighted Article:** This work provides the first molecular description of dynamic cellular reprogramming during haustorium development in parasitic plants by live imaging-based marker expression and cell lineage analyses.

## INTRODUCTION

Parasitic plants have evolved independently at least 12 times in angiosperms, and ∼4500 parasitic species are known to date (Barkman et al., 2007; Westwood et al., 2010; Yoshida et al., 2016). Orobanchaceae is the most species-rich family of parasitic angiosperms and most members of this family are either facultative or obligate parasites, except for the nonparasitic genus *Lindenbergia* (Olmstead et al., 2001). Notably, *Striga* spp. and *Orobanche* spp. are devastating agricultural pests, infecting staple food crops (Parker, 2009; Spallek et al., 2013). Their seeds can be extremely small and are produced in large quantities, making eradication of *Striga* spp. and Orobanche spp. difficult (Spallek et al., 2013).

To obtain water and nutrients from their hosts, parasitic Orobanchaceae invade host root vasculatures and establish a physiological connection via a specialized inducible organ called a haustorium. The haustorium can be classified as lateral or terminal, depending on its developmental origin. The former develops laterally from the primary root of a facultative parasite, or from secondary roots of both facultative and obligate parasites. By contrast, the terminal haustorium is formed only in obligate parasites and develops at the radicle tip, attaching to the host immediately after germination to secure the sole nutrient source for the parasite during its early development. During Orobanchaceae evolution, the lateral haustorium is thought to have arisen concomitantly with parasitism. Terminal haustoria appear to have occurred with the subsequent independent evolution of obligate parasitism (Westwood et al., 2010).

Most Orobanchaceae initiate haustorium development upon sensing external chemical cues derived from host-produced compounds, collectively called haustorium-inducing factors (HIFs). For example, 2,6-dimethoxy-1,4-benzoquinone (DMBQ) is an HIF originally isolated from the root extracts of sorghum, a natural host for several *Striga* spp. (Chang and Lynn, 1986). DMBQ is also a potent trigger of haustorium organogenesis in facultative Orobanchaceae parasites, such as *Agalinis purpurea* (Baird and Riopel, 1984), *Triphysaria versicolor* (Albrecht et al., 1999) and *Phtheirospermum japonicum* (Ishida et al., 2016, 2017). Upon exposure to HIFs or host roots, haustorium organogenesis begins with the radial enlargement of cortical layers followed by anticlinal divisions in the root epidermis, which establish the haustorium apex (Baird and Riopel, 1984). During this early stage, haustorial hairs, which facilitate physical interaction with host plants, also begin to differentiate from epidermal cells (Baird and Riopel, 1984; Cui et al., 2016).

The specific cells that develop at the haustorium interface with the host are called intrusive cells, and have distinctive morphological features (Musselman and Dickison, 1975). These cells are highly elongated and, based on electron microscopic analysis in *T. versicolor* (Heide-Jørgensen and Kuijt, 1993), potentially originate from the epidermis. Currently known HIFs are not able to induce intrusive cells, indicating that another host factor (or factors) is required for induction (Estabrook and Yoder, 1998). After intrusive cells reach host vascular tissues, portions of adjacent haustorial cells differentiate into tracheary elements, forming a connective xylem bridge between the parasite and host root vascular systems. Although such xylem-vessel connections are common, phloem connections between an Orobanchaceae parasite and a host have been reported only in the obligate parasites *Alectra vogelii* and *Orobanche crenata* (Dörr et al., 1979; Zhou et al., 2004).

Despite the number of early microscopic studies, the developmental origin of cells in haustoria remains obscure. One potential way to produce a new organ in the root is to generate a primordium from pericycle founder cells with stem cell activity, as seen in lateral root development in *Arabidopsis thaliana* (Malamy and Benfey, 1997). In this case, either individual or pairs of meristematic pericycle founder cells undergo anticlinal divisions and then start to divide periclinally to create a dome-shaped primordium (Laskowski et al., 1995; Malamy and Benfey, 1997). However, unlike lateral root development, there has been no report of meristematic pericycle founder cells being the source of haustorial cells. Alternatively, it is possible that more differentiated cells (i.e. epidermal, cortex or endodermal cells) divide and change their cell identity. In this case, those cells need to be coordinately reprogrammed to be able to generate a functional organ.

To understand the molecular mechanism of *de novo* organogenesis, we utilized *P. japonicum*, a model for studying parasite-host molecular interactions (Cui et al., 2016; Ishida et al., 2011, 2016; Spallek et al., 2017). Here, we describe dynamic cell fate transitions during lateral haustorium development in *P. japonicum* using live-imaging to determine expression patterns of cell type-specific marker genes. In addition, clonal analysis of cell lineages revealed that cells the fate of which was already determined reprogram their identities to become procambium-like cells, which further differentiate into tracheary elements for xylem bridge formation. These results provide the first cell fate transition map of induced cellular reprogramming during haustorium organogenesis.

## RESULTS

### Dynamics of tissue reorganization and cell division during haustorium organogenesis

**Fig. 1.**
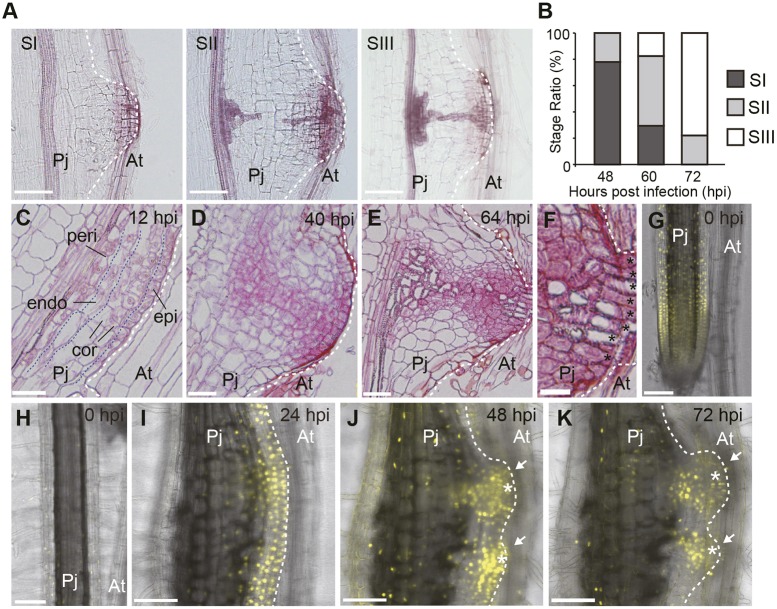
**Lateral haustorium structure at different developmental stages of *Phtheirospermumjaponicum*.** (A) Developmental stages of the xylem bridge were classified into three categories: SI, pre-initiation; SII, developing; and SIII, fully connected. (B) Ratio of each developmental stage at the indicated time point. *n*=21∼26 for each time point. (C-E) Longitudinal sections of haustorium at the indicated time point. Blue broken lines delineate the layer of indicated cell types: epi, epidermis; cor, cortex; endo, endodermis; peri, pericycle. Sections were stained with Safranin-O. (F) Magnified view of the interface region of the haustorium indicated in E. Black asterisks indicate intrusive cells. (G-K) Expression pattern of the *AtCYCB1;2* promoter in the *P. japonicum* root meristematic region (G) and differentiated region (H) before haustorium formation, and during haustorium development at the indicated time points (I-K). Six out of eight hairy roots showed a similar expression pattern. The same haustorium is shown in I-K. Two haustoria developed in close proximity, indicated by white arrows. White asterisks denote intrusive cells. YFP fluorescence is in yellow. Bright-field and YFP fluorescent images are merged. Scale bars: 100 μm in A,G-K, 50 μm in C-E, 20 μm in F. Pj, *P. japonicum* root; At, *Arabidopsis thaliana* root.

To investigate haustorium development at the molecular level, we first established a robust and synchronized method for *in vitro* haustorium induction using *P. japonicum* as the parasite and *A. thaliana* as the host (see Materials and Methods). Haustoria were induced in a highly synchronous manner (Fig. 1A,B). Xylem bridge formation was visualized with Safranin-O staining and used as an indicator of different developmental stages (Fig. 1A). At Stage I, there were no tracheary elements in the haustorium. At Stage II, tracheary elements were differentiated near the main xylem of the parasite and near the tissues that attached to the host. At Stage III, the xylem connection was established. Almost 80% of infecting plants were at Stage I at 48 h post infection (hpi), whereas almost 80% of *P. japonicum* roots had established xylem connections with host roots (Stage III) at 72 hpi (Fig. 1B). To observe the detailed tissue structure, we prepared thin longitudinal sections of induced haustoria at various developmental time points (Fig. 1C-E). At 12 hpi, cell divisions were observed in all cell layers, from the outermost epidermis to the pericycle, at the side facing the host (Fig. 1C). At 40 hpi, a dome-shaped structure was formed facing toward the host root (Fig. 1D). At 64 hpi, a new tissue pattern was visible in the haustorium, with small cells located in the central area that subsequently differentiated into tracheary elements. In addition, intrusive cells developed at the interface with host roots at this time point (Fig. 1E,F).

To identify where cell division occurs during haustorium formation, we monitored Yellow Fluorescent Protein (YFP) expression under the control of the *A. thaliana CYCB1;2* promoter, a general cell division marker expressed during the mitotic phase (Ishida et al., 2011; Ito, 2000). This marker was expressed strongly in the meristematic region of the root tip before haustorium induction (Fig. 1G), but not in the differentiated region (Fig. 1H), indicating that it can also be used as a cell cycle marker in *P. japonicum*. At 24 hpi, YFP signals were observed from the root tissues on the side adjacent to the host, but not on the other side of the root (Fig. 1I). At 48 hpi, YFP signals were detected at the apex and the central region of the forming haustorium (Fig. 1J). At 72 hpi, YFP signals were observed in tissue near the intrusive region, whereas weak signals were detected from intrusive cells (Fig. 1K). These expression patterns were consistent with the pattern of cell division observed in our time-lapsed longitudinal sections of haustoria (Fig. 1C-E).

To gain insight into the formation of the dome-shaped structure of haustoria, we tracked the direction of cell division across multiple cell layers during the early phase of haustorium development by observing nuclear behavior under a confocal microscope (Fig. S1A-C, Movie 1). Sequential confocal images of nuclear-localized green fluorescent protein (GFP) signals were taken for 14 h (6∼20 hpi) in transgenic *P. japonicum* roots carrying the *AtRPS5a::H2B-GFP* construct (Fig. S1A) (Adachi et al., 2011). Nuclear movement and cell lineage were tracked semiautomatically using the Trackmate software (Tinevez et al., 2016). Interestingly, only anticlinal divisions were observed in the outer layers, including the epidermis, cortex and endodermis (Fig. S1B, Movie 2), indicating that outer tissues preserve their layer morphology during the early developmental phase. By contrast, periclinal divisions were observed in stele tissues (Fig. S1C, Movie 3). These cell division patterns could give rise to the dome-shaped structure of haustoria.

### Expression dynamics of cell type-specific markers during haustorium development

**Fig. 2.**
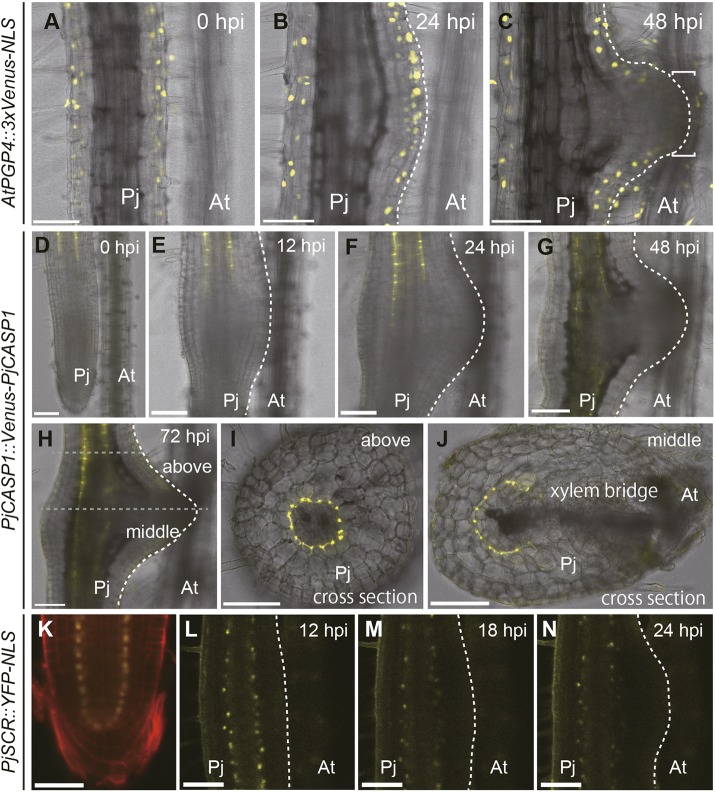
**Expression dynamics of epidermis and endodermis markers during haustorium development.** (A-C) Expression pattern of the *AtPGP4* promoter as an epidermis marker in *Phtheirospermum* *japonicum* during haustorium development at the indicated time points. All four hairy roots showed a similar expression pattern. Square brackets in C denote the intrusive region. Bright-field and Venus fluorescent images are merged. (D-J) The expression pattern of *PjCASP1::Venus-PjCASP1* in *P. japonicum* during haustorium development at the indicated time points. All six hairy roots showed a similar expression pattern. Bright-field and Venus fluorescent images in yellow are merged. I and J show cross-sections of the top and mid-regions of the haustorium shown in H, respectively. Gray-dotted lines indicate the position of sections. (K-N) Expression pattern of the *PjSCR* promoter in *P. japonicum* root tip (K) and developing haustorium at the indicated time points (L-N). (K) Propidium iodide staining is in red. (L-N) Bright-field and YFP fluorescent images are merged. The same haustoria are shown in A-C, D-J, and L-N. Scale bars: 100 μm. Pj, *P. japonicum* root; At, *Arabidopsis* *thaliana* root.

To analyze cellular reprogramming during haustorium formation further, we established various cell type-specific markers and investigated their expression patterns. As an epidermis marker, we utilized the *AtPGP4* promoter to express a YFP variant, Venus, fused with a nuclear localization signal (NLS) (*AtPGP4::3xVenus-NLS*) (Ishida et al., 2016; Nagai et al., 2002; Terasaka et al., 2005). Before and during early haustorium development (∼24 hpi), Venus-derived fluorescence was specifically observed in epidermis, including hair cells, thus confirming its cell specificity (Fig. 2A,B). Interestingly, at 48 hpi, the Venus fluorescence disappeared in the outermost cells at the haustorium apex that was adjacent to the host root, suggesting the loss of epidermal identity in these cells, which eventually became intrusive cells (Fig. 2C). Meanwhile, epidermal cells in other parts of the haustorium retained the marker expression (Fig. 2C), indicating that this cell fate transition is specific to the intrusive cells.

As an endodermis marker, we tested a putative homolog of *CASPARIAN STRIP MEMBRANE DOMAIN PROTEIN 1* (*CASP1*) and *SCARECROW* (*SCR*) in *P. japonicum*, designated as *PjCASP1* (Fig. S2) and *PjSCR* (Fig. S3), fused with *Venus* (*PjCASP1::Venus-PjCASP1*) and nuclear-localized YFP (*PjSCR::YFP-NLS*), respectively (Di Laurenzio et al., 1996; Roppolo et al., 2011). Given that CASP proteins are normally required for Casparian strip formation, their expression should be tightly localized to the Casparian strip-originating tissues. Indeed, the Venus fluorescence derived from *PjCASP1::Venus-PjCASP1* was specifically observed in the endodermal layer above the elongation zone, which surrounded stele tissues, as reported in *A. thaliana* (Roppolo et al., 2011) (Fig. 2D). However, when haustorium formation was initiated, the elongation zone close to host roots did not exhibit Venus fluorescence (Fig. 2E,F). This alteration at the haustorium-emerging site continued even after xylem bridge connection, resulting in a disjointed expression pattern of PjCASP1 in the haustorium (Fig. 2G,H). Cross-sectional imaging revealed that PjCASP1 protein expression surrounded normal stele tissue above the haustorium at 72 hpi (Fig. 2I), whereas its expression was interrupted by the xylem bridge in the middle part of the haustorium (Fig. 2J). *PjSCR* promoter expression was detected in the quiescent center (QC), endodermis and/or cortex initials and the endodermis layer in the *P. japonicum* root tip, and this expression pattern was similar to that of *AtSCR* in the *A. thaliana* root tip (Fig. 2K) (Di Laurenzio et al., 1996). *PjSCR* promoter expression gradually decreased during haustorium development because the distance between these tissues and the meristematic region gradually increased. Nevertheless, we observed an apparent reduction in *PjSCR* expression specifically in the haustorium initiation site, similarly to PjCASP1 expression (Fig. 2L-N). To clarify whether Casparian strips form in haustoria, we visualized Casparian strips using Basic Fuchsin (Ursache et al., 2018). We confirmed that Basic Fuchsin staining visualized Casparian strips in the endodermis and the exodermis (the outer cortex layer) in normal *P. japonicum* roots (Fig. S4A,B). We also found that Casparian strip formation in endodermis essentially followed the expression pattern of *PjCASP1* (Fig. S4C-E). In addition to Casparian strips in the endodermis and the exodermis, lignin deposition was also detected in the border surrounding xylem bridge tissues. This might work as an apoplastic barrier to secure nutrient transfer from the host plants. These observations suggest that endodermal cells are missing in the middle of haustoria and that other types of cell occupy this space.

**Fig. 3.**
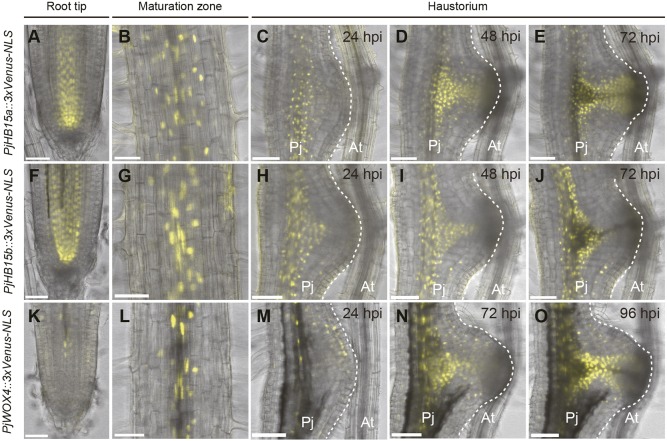
**Expression dynamics of putative procambium genes during haustorium development.** Expression pattern of *PjHB15a::3xVenus-NLS* (A-E), *PjHB15b::3xVenus-NLS* (F-J) and *PjWOX4::3xVenus-NLS* (K-O). Expression patterns in root tip and maturation zone and during haustorium formation are shown as indicated. The broken white lines mark the edge of the haustorium. Venus fluorescence is in yellow. Bright-field and Venus fluorescent images are merged. The same haustoria are shown in each time-course observation in C-E, H-J and M-O. Two out of two, three out of four, and five out of six hairy roots showed a similar expression pattern, as shown in A-E, F-J and K-O, respectively. Scale bars: 50 μm in A,B,F,G,K,L; 100 μm in C-E, H-J, M-O. Pj, *Phtheirospermum* *japonicum* root; At, *Arabidopsis* *thaliana* root.

Next, we tested *P. japonicum* homologs of *A. thaliana* genes [*ARABIDOPSIS HOMEOBOX PROTEIN 15* (*AtHB15*), *AtHB8* and *WUSCHEL-RELATED HOMEOBOX 4* (*WOX4*)] that are expressed in procambium (Hirakawa et al., 2010; Ji et al., 2010; Ohashi-Ito and Fukuda, 2003; Prigge et al., 2005). Two putative *AtHB15* homologs, designated *PjHB15a* and *PjHB15b*, were found in the *P. japonicum* genome (Fig. S5) and their promoter regions were cloned to drive the expression of triple Venus with NLS (*PjHB15a::3xVenus-NLS* and *PjHB15b::3xVenus-NLS*, respectively). In the absence of a host, *PjHB15a* and *PjHB15b* were strongly expressed in vascular initials above the QC, and weakly expressed in vascular tissues (Fig. 3A,B,F,G). As haustorium development progressed in the presence of the host, *PjHB15a* and *PjHB15b* were strongly expressed in the central region of haustoria before xylem bridge formation (Fig. 3C,D,H,I). At 72 hpi, *PjHB15a* expression remained around the newly formed xylem bridge (Fig. 3E), whereas *PjHB15b* expression was significantly reduced compared with that of *PjHB15a* (Fig. 3J).

One putative *AtHB8* homolog, designated *PjHB8*, was found in the *P. japonicum* genome (Fig. S5) and its promoter region was cloned to drive triple Venus fused to *Arabidopsis* SYNTAXIN OF PLANTS (SYP) protein, which localizes to the plasma membrane (*PjHB8::3xVenus-SYP*) (Uemura et al., 2004). In the absence of host, *PjHB8* was expressed in almost all cell types in the root tip, whereas, in the other region of the root, its expression was predominantly observed in stele tissues (Fig. S6A,B). However, in the haustorium, the *PjHB8* expression pattern was similar to that of *PjHB15*a and *PjHB15b* (Fig. S6C-E). Notably, *PjHB8* expression was also visible in the intrusive cells (Fig. S6E). Cross-sectional analyses of the haustorium also showed that *PjHB8*-positive cells were relatively small and located along the xylem bridge (Fig. S4F).

Only one *WOX4* homolog, *PjWOX4*, was found in the *P. japonicum* genome (Fig. S7). *PjWOX4* expression (*PjWOX4::3xVenus-NLS*) was specifically observed in the root vascular tissues, but was absent in the meristematic region (Fig. 3K,L), similar to *AtWOX4* expression pattern in *A. thaliana* roots (Hirakawa et al., 2010). During early haustorium development, *PjWOX4* promoter activity was detected at the haustorium apex and this expression was similar to that in the auxin-responsive region (Fig. 3M) (Ishida et al., 2016). At 72 hpi, its expression became similar to that of *PjHB15a* (Fig. 3N). After xylem bridge formation at 96 hpi, its expression became stronger in the cells surrounding the xylem bridge, and was similar to that of *PjHB15a* (Fig. 3O).

**Fig. 4.**
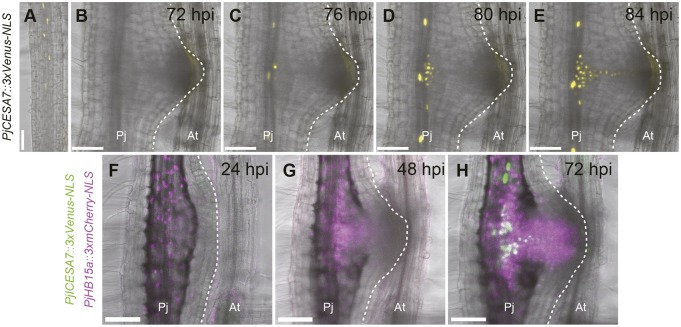
**Expression dynamics of the xylem cell marker *PjCESA7* during haustorium development.** *PjCESA7* expression dynamics in (A) root tip and during haustorium formation (B-E) at the indicated time points. Six *Z*-stack photos (43 μm thickness in total) were stacked using the maximum projection method. Venus fluorescence is in yellow. Bright-field and Venus fluorescent images are merged. Four out of five hairy roots showed a similar expression pattern. (F-H) Expression pattern of *PjCESA7::3xVenus-NLS* and *PjHB15a::3xmCherry-NLS* in the same haustorium at the indicated time points. Venus fluorescence is in green. mCherry fluorescence is in magenta. Bright-field, Venus fluorescent and mCherry fluorescent images are merged. The broken white lines mark the edge of the haustorium. Two out of two hairy roots showed a similar expression pattern. The same haustoria are shown in each time-course observation in B-E and F-H. Scale bars: 100 μm. Pj, *Phtheirospermum* *japonicum* root; At, *Arabidopsis* *thaliana* root.

Next, we tested a homolog of *A. thaliana CELLULOSE SYNTHASE CATALYTIC SUBUNIT 7* (*CESA7*, also known as *IRREGULAR XYLEM 3*) (Fig. S8). *AtCESA7* encodes a xylem-specific cellulose synthase for secondary cell wall synthesis and is expressed in tracheary elements (Mitsuda et al., 2007; Taylor et al., 1999). When the host was absent, *PjCESA7* expression (observed with *PjCESA7::3xVenus-NLS*) started in the root differentiation zone during protoxylem formation, as previously described for *AtCESA7* in *A. thaliana* (Fig. 4A) (Taylor et al., 1999). At 76 hpi, *PjCESA7* began to be expressed before xylem bridge formation in the haustorium (Fig. 4B-E) and its expression followed the xylem bridge pattern. In addition, *PjCESA7* expression was also observed in the root vascular cells, likely reflecting metaxylem differentiation (Fig. 4C-E). *PjCESA7* was induced in cells that already expressed *PjHB15a*, as also observed in the double-marker analysis using *PjCESA7::3xVenus-NLS* and *PjHB15a*::3xmCherry-NLS simultaneously (Fig. 4F-H). Procambium cells differentiate into not only xylem cells, but also phloem cells. To assess whether phloem tissues are developed in haustoria, we utilized the *Arabidopsis ALTERED PHLOEM DEVELOPMENT* (*AtAPL*) promoter, which is expressed in phloem (Bonke et al., 2003). We confirmed that the *AtAPL* promoter was expressed in protophloems in *P. japonicum* roots, as also reported for *A. thaliana* (Fig. S9A) (Ursache et al., 2018). We tracked the expression pattern of the *AtAPL* promoter in haustoria for 7 days after infection, and did not observe any signals (Fig. S9B-E). These results indicated that procambium cell marker (*PjHB15*, *PjHB8* and *PjWOX4*)-positive cells (hereafter denoted as procambium-like cells) initially develop in the central and the intrusive regions in haustoria. Some of these cells subsequently differentiate into tracheary elements to form xylem bridges.

### Clonal analyses of root cell lineages during haustorium development

**Fig. 5.**
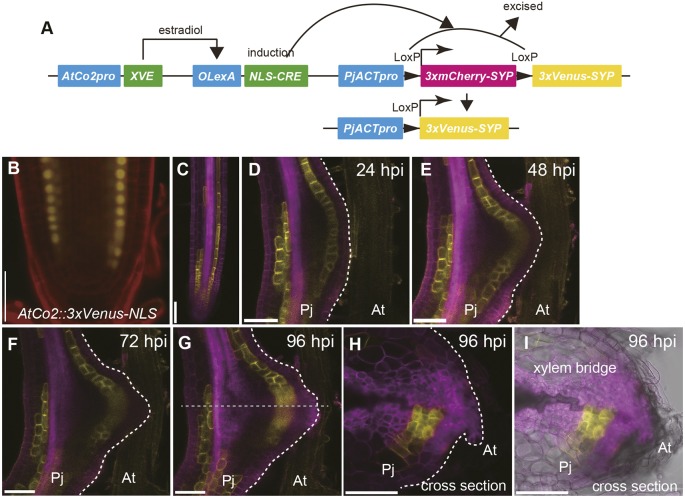
**Cell lineage analysis of inner cortex during haustorium development.** (A) Schematic view of the estradiol-inducible CRE-Lox system for cell lineage tracking. (B) Expression pattern of *AtCo2::3xVenus-NLS* in *Phtheirospermum* *japonicum* root. (C) Estradiol treatment induced the expression of Venus fluorescent protein in inner cortex. (D-I) Expression pattern of Venus fluorescent protein in inner cortex lineage during haustorium formation at the indicated time points. (H,I) Cross-section of the haustorium shown in G. Broken gray line indicates the position of the cross-section. Venus fluorescence is in yellow. mCherry fluorescence is in magenta. PI staining is in red. Venus fluorescent and PI staining images are merged in B. Venus fluorescent and mCherry fluorescent images are merged in C-H. Venus fluorescent, mCherry fluorescent and bright-field images are merged in I. Two out of two hairy roots showed a similar result. The same haustorium is shown in D-I. Scale bars: 50 μm in B, 100 μm in C-I. Pj, *P. japonicum* root; At, *Arabidopsis* *thaliana* root.

To further investigate the contribution of each cell type to haustorium development, we performed clonal analyses and followed the lineages of various cell types. Here, we utilized the CRE-Lox system because it can selectively target specific cell types for lineage analysis by using cell type-specific promoters. We developed an estradiol-inducible CRE-Lox system (Fig. 5A), in which estradiol treatment activates the XVE transactivator expressed in a specific cell type, thus triggering cell type-specific CRE recombinase expression. Subsequently, CRE recombinase excises the floxed mCherry sequence, the expression of which is driven under actin promoter (PjACTpro), switching fluorescent protein expression from mCherry to Venus. Therefore, specific cell types can be clonally marked with the Venus protein. The *AtCo2* promoter (*AtCo2>>NLS-CRE*) (Heidstra et al., 2004), which was specifically expressed in the inner cortex of *P. japonicum* roots (Fig. 5B), was used to follow the cortical lineages. Although not all inner cortex cells expressed Venus upon CRE recombinase induction after 24 h of estradiol treatment (Fig. 5C), transformants with sufficient recombination events were available for the lineage analysis. At 24 hpi, fluorescent inner cortical cells were visible as a single cell layer in the haustorium (Fig. 5D). In contrast to earlier developmental stages where only anticlinal cell divisions were observed (Fig. S1B), periclinal divisions were observed in the inner cortex lineage at 48 dpi, resulting in a double cell layer in the inner cortex (Fig. 5E). The inner cortical cells retained their relative position within a haustorium, neither penetrating the outer layers nor being penetrated by the inner layers (Fig. 5F-I). In the haustorium cross-section at 96 hpi, Venus fluorescence was detected in the procambium-like cells around the xylem bridge (Fig. 5H,I). These data suggest that the cortex cells change their identity to become procambium-like cells in haustoria.

**Fig. 6.**
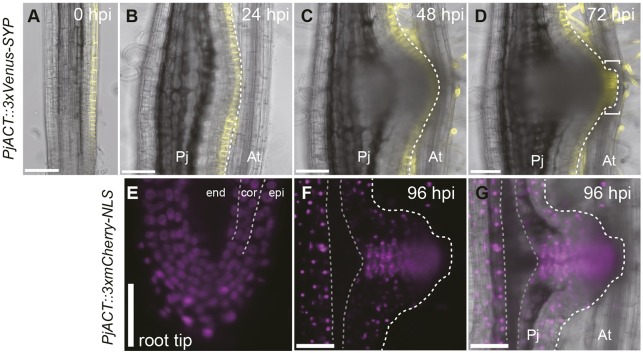
**Cell lineage analysis of epidermis and stele tissue during haustorium development.** (A) Transformed root chimerically expressing Venus fluorescent protein in several cell files in epidermis only. (B-D) Epidermal cell lineage during haustorium formation. Square brackets indicate the intrusive region. Venus fluorescence is in yellow. Bright-field and Venus fluorescent images are merged. (E) Transformed root chimerically expressing mCherry fluorescent protein in epidermis (epi), endodermis (end) and cortex (cor). (F,G) Cell lineage of mCherry-expressing cells in haustorium at 96 hpi. (G) Fluorescent image in F is merged with a bright-field image. The broken white lines mark the edge of the haustorium. Broken gray lines mark mCherry-negative tissue. The same hairy roots are shown in A-D and E-G. Scale bars: 100 μm. Pj, *Phtheirospermum* *japonicum* root; At, *Arabidopsis* *thaliana* root.

We also utilized transformed roots chimerically expressing the triple Venus with SYP only in epidermal cells (Fig. 6A). In this particular hairy root, the Venus fluorescent signal remained in the outermost epidermal layer at 24 and 48 hpi (Fig. 6B,C). Interestingly, at 72 hpi, intrusive cells that expressed Venus signals were observed (Fig. 6D). These data indicate that intrusive cells originate from the epidermal cells. Given that intrusive cells lost the epidermis marker (Fig. 2C) and there was no evidence of inner cells penetrating the epidermal layers, we concluded that the cell identity had changed from epidermal to intrusive cells or their precursor cells. Likewise, we analyzed the other transformed root lines that expressed nuclear-localized mCherry protein (*PjACT::3xmCherry-NLS*) only in the endodermis, cortex and epidermis but not in cells in stele tissues (Fig. 6E), and followed these cell lineages during haustorium development. At 96 hpi, after xylem bridge formation, mCherry fluorescence was detected in most procambium-like cells, (Fig. 6F,G), suggesting that procambium-like cells in haustoria were mainly derived from endodermis, cortex or epidermis, but not from root stele tissues. Thus, we concluded that the cell identity was changed to generate new vascular tissues in haustoria.

## DISCUSSION

### Cell cycle re-entry in multiple cell types

In this study, we captured dynamic cell fate transitions during haustorium development using live-imaging techniques in the Orobanchaceae facultative parasite *P. japonicum*. At the haustorium initiation site, cell division began at almost the same time in different root cellular layers (Fig. 1C,I), with epidermal, cortex and endodermal cells exhibiting anticlinal initial division, and pericycle cells showing periclinal initial division (Fig. S1B,C). This is in stark contrast to other organ initiation processes in root, such as lateral roots and nodules, where anticlinal cell division in the pericycle initiates organ development (Laskowski et al., 1995; Malamy and Benfey, 1997; Xiao et al., 2014). Given that haustoria originate from relatively young tissues near the meristematic region compared with lateral roots and nodules (Bhuvaneswari et al., 1980; Parizot et al., 2008), not only pericycle cells, which maintain stem cell activity in mature root tissue, but also other cell types might retain the potential to divide upon haustorium induction.

### Involvement of epidermis in haustorium formation

Another uniqueness of haustorium development is the involvement of epidermal cells in haustorium formation. In *A. thaliana*, lateral root originates from the pericycle (Dolan et al., 1993). In other species, endodermal and cortex layers were also reported to be involved in lateral root formation and to become part of a new lateral root (Casero et al., 1995; Mallory et al., 1970). In the case of nodule formation, although the epidermis functions to perceive nodulation signals and acts as an entry site for rhizobium infection, it is not actively involved in tissue restructuring per se (Gage, 2004; Xiao et al., 2014). In contrast to these types of organogenesis, epidermal cells vigorously divide from the very early stages of haustorium development (Fig. 1C,I). Consistently, a key auxin biosynthesis enzyme, YUC3, is upregulated specifically in the epidermis upon host perception, resulting in high auxin response and, thus, reactivation of cell division at the haustorium initiation site (Ishida et al., 2016). Interestingly, epidermal cell division upon haustorium induction has also been observed in the stem parasite *Cuscuta* spp. (Lee, 2008).

During later developmental stages, intrusive cells develop at the parasite-host interface during host tissue invasion (Kuijt, 1969). Our cell lineage analysis clearly showed that intrusive cells originate solely from the epidermis (Fig. 6D). Expression of the epidermis marker disappeared at the haustorium apex upon host tissue invasion at ∼48 hpi (Fig. 2C), indicating that a transition from epidermal cells to intrusive cells occurred around this period. However, it is still unclear whether intrusive cells themselves continuously proliferate or the meristematic region for intrusive cells, if it exists, continuously supplies intrusive cells until they reach host vasculature. In the case of *Cuscuta* spp*.*, cells in meristem for the intrusive region, described as endophyte primordium, proliferate and differentiate into searching hyphae, which eventually connect to host vasculature (Lee and Lee, 1989). In *P. japonicum*, cells in the intrusive region initially showed transient division (Fig. 1J). At the later stage where most cells in the haustorium apex have already stopped undergoing cell division, cell division was detected only in the region in the vicinity of the intrusive region, but not in the intrusive region itself (Fig. 1K). This region might function similarly to the endophyte primordium in *Cuscuta* haustoria. A drawback in our experimental system is that it was difficult to assess whether intrusive cells are capable of continuously dividing until they reach the host vasculature. This is because of the very small diameter of the *A. thaliana* root. Therefore, we need to use an alternative host with larger roots, such as rice, to investigate this aspect.

### Development of procambium-like cells for xylem bridge formation

Cambium-like cells are assumed to be the source of tracheary elements required for xylem bridge formation and, thus, host connection in Orobanchaceae parasitic plants (Musselman and Dickison, 1975). A previous report showed that procambium-like strands surround the xylem bridge cylindrically in the haustorium of the facultative Orobanchaceae parasite *Buchnera hispida* (Neumann et al., 1999). In our analysis, we found that four procambium marker genes were expressed in the cells surrounding the xylem bridge. Given where these genes are expressed, as well as the conserved function of *WOX4* in stem cell maintenance in *A. thaliana* and rice, these genes might act in concert to maintain the meristematic activity of particular cells in the central region of haustoria. We designated these cells as procambium-like cells, which eventually establish a *de novo* vasculature connection to the host. Indeed, *PjHB15a* expression preceded that of *PjCESA7* during haustorium development (Fig. 4F-H), demonstrating that tracheary elements that form a xylem bridge toward the host are differentiated from procambium-like cells. Interestingly, the number of the xylem bridge connection to host vasculature increases as haustoria develop (Spallek et al., 2017). This situation is similar to secondary root growth, where cambium produces xylem. Similarly, cytokinin, a positive regulator of cambial activity (Matsumoto-Kitano et al., 2008), is produced in significant quantities and haustoria show a strong cytokinin response (Spallek et al., 2017). However, cambium also normally produces phloem, although this is not the case for *P. japonicum* haustoria (Spallek et al., 2017). Thus, procambium-like cells in haustoria might be distinct from procambium cells. Alternatively, it is possible that cues to form xylem, but not phloem, originate from the host. Supporting this idea, haustoria induced in the absence of a host plant using only HIFs are unable to induce xylem formation (Estabrook and Yoder, 1998). To define the identity of these procambium-like cells in haustoria, further single cell-type expression analyses will be required.

### Cellular reprogramming to procambium-like cells

**Fig. 7.**
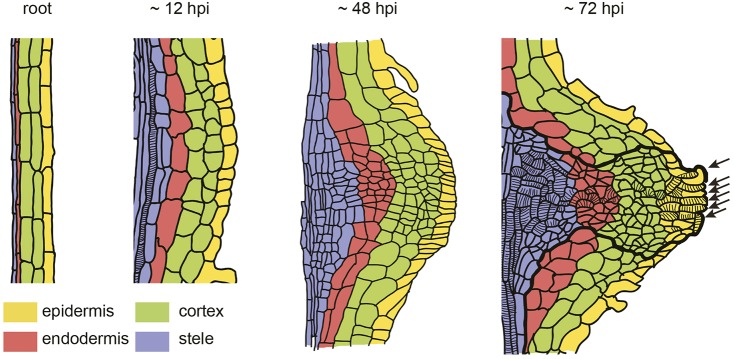
**Cell fate transition map during *de novo* haustorium organogenesis.** Schematic representations of haustorium organization at indicated time points. One side of the root is described. The origins of cell types are color coded as shown. Cells inside the thick line represent procambium marker-positive cells in later stages. Stripes mark tracheary elements. Arrows indicate intrusive cells and intrusive cell-derived tracheary elements.

Our cell lineage analyses unequivocally demonstrated cell fate transition during haustorium development. For instance, cells that expressed the inner cortex marker before host infection were present in the haustorium central region, where procambium markers were expressed (Fig. 5G-I), indicating that inner cortex cells have transitioned to procambium-like cells. In addition, original stele tissues in the root contributed only toward the basal part of haustoria (Fig. 5E-G). Moreover, no CASP1 expression was observed upon haustorium induction, specifically at the haustorium emergence site, suggesting that the modulation of endodermal cell identity occurs during the early stage of haustorium development (Fig. 2F). Based on these results, we propose a dynamic cell fate transition map where procambium-like cells differentiate from multiple cell types during *de* *novo* haustorium organogenesis (Fig. 7). It is interesting that the cell fate transition to procambium-like cells only occurs in the haustorium central region but not in the peripheral region. Host-derived signals other than HIFs are required for the vascular formation in haustoria (Estabrook and Yoder, 1998). Thus, as yet unknown host-derived signals might contribute to the local induction of cell fate transitions within the haustorium.

## MATERIALS AND METHODS

### Chemicals and reagents

All chemicals were purchased from Wako (Japan) unless otherwise stated.

### Plant materials and growth conditions

*P. japonicum* (Thunb.) Kanitz seeds were handled as previously described (Ishida et al., 2011; Yoshida and Shirasu, 2009). The *A. thaliana* Col-0 ecotype was used as the host to induce haustorium in *P. japonicum*. For *in vitro* germination, *P. japonicum* seeds were surface sterilized with 10% (v/v) commercial bleach solution (Kao) for 5 min, followed by five rinses with sterilized water. Seeds were then sown on half-strength Murashige and Skoog (MS) solid medium [0.8% (w/v) agar (Bacto), 1% (w/v) sucrose]. After overnight stratification at 4°C in the dark, plants were grown, either vertically for infection assay on plates or horizontally for transformation, at 25°C under long-day conditions (16 h light/8 h dark). *A. thaliana* plants were germinated after sterilization (70% ethanol for 10 min) and stratification at 4°C in the dark, and grown on half-strength MS solid medium (0.8% agar, 1% sucrose) at 22°C under long-day conditions.

### *In vitro* infection assay

Ten- to 12-day-old *P. japonicum* seedlings were transferred to 0.7% INA agar plate (Funakoshi) without any nutrients and were grown for a further 2 days vertically at 25°C under long-day conditions. Seven- to 10-day-old *A. thaliana* seedlings were carefully placed next to *P. japonicum* seedlings so that the meristematic zone of the *P. japonicum* root made contact with the *A. thaliana* root. Plants were maintained vertically during the infection period at 25°C under long-day conditions.

### Histological staining

For Safranin-O staining, haustoria were excised from roots and incubated in 10% (w/v) KOH at 90°C for 15 min. After three rinses with PBS buffer (137 mM NaCl, 2.7 mM KCl, 10 mM Na_2_HPO_4_ and 2 mM KH_2_PO_4_, pH 7.4 adjusted by HCl), samples were incubated in 0.1% Safranin-O (w/v) (Sigma), 30% ethanol solution at 90°C for 5 min. Samples were then rinsed three times with PBS buffer and cleared with clearing solution (8 g chloral hydrate, 1 ml glycerol and 2 ml water) overnight.

For visualization of Casparian strips and cell wall, 7-day-old *P. japonicum* roots and haustoria, and hairy roots were stained with 0.2% Basic Fuchsin (Sigma) and 0.1% Calcofluor White (Sigma), and 0.1% Direct Red 23 (Sigma), respectively, according to a previously published paper (Ursache et al., 2018).

### Tissue embedding, sectioning and staining

Haustoria were separated from roots and were vacuum treated in FAA solution [10% formaldehyde, 5% acetic acid and 50% ethanol (v/v)] for 15 min followed by 2-h incubation at room temperature. Samples were subjected to a series of dehydration with ethanol and embedded into Technovit 7100 resin (Heraeus Kulzer GmbH) according to the manufacturer's instruction. Solidified resin blocks were sectioned using a microtome (Leica, RM2135) to 4 μm thickness. Sections were stained with 0.1% Safranin-O dissolved in water and then washed with water several times.

### Phylogenetic analysis

Phylogenetic analysis was performed using MEGA6 (Tamura et al., 2013). Full amino acid sequences were aligned using the ClustalW program. Phylogenetic trees were drawn using the maximum-likelihood method with a bootstrap value of 100.

### Plasmid construction

For plasmid construction, Golden Gate cloning technology was used unless otherwise stated (Engler et al., 2014). All restriction sites for *Bpi*I and *Bsa*I restriction enzymes were mutated. All primers used in this study are listed in Table S1. Four fluorescent marker modules (3xVenus-SYP, 3xmCherry-NLS 3xVenus-NLS and 3xmCherry-SYP) were assembled into pICH41308 level 0 as previously described (Cui et al., 2016). Promoter sequences of *PjHB15a*, *PjHB15b*, *PjWOX4*, *PjACT*, *PjCASP1* and *AtAPL* (GenBank accession numbers LC363587, LC363588, LC363590, LC363592 and LC363593, respectively) were PCR amplified from *P. japonicum* genomic DNA and *A. thaliana* (Col-0) genomic DNA. Given that these sequences were large, each of these gene promoter regions was cloned separately as two-part fragments into a pAGM1311 level –1 vector. They were then combined into pICH41295 level 0 vectors individually. *PjCESA7* and *AtCo2* promoter sequences (*PjCESA7*; GenBank accession number LC363591) were PCR amplified from genomic DNA and directly cloned into pICH41295 level 0 vectors. These promoter modules were further combined with one of the four fluorescent marker modules into a level-1 vector, which was then used to generate a level-2 pAGM4723 vector without any additional sequence for *Agrobacterium*-mediated hairy root transformation. For double-marker analyses, *PjCESA7::3xVenus-NLS* and *PjHB15a::3xmCherry-NLS* were combined into a level-2 pAGM723 vector. To obtain *PjCASP1::Venus-PjCASP1*, the PjCASP1 coding region and 3′-untranslated region (UTR) regulatory sequence (GenBank accession number LC363593) were PCR amplified from genomic DNA and cloned into pICH41264 and pICH41276 level 0 vectors, respectively. One repeat of the Venus DNA sequence was cloned into a pICH41258 level 0 vector. These vectors, together with the *PjCASP1* promoter module, were combined into a level-1 vector.

To generate an estradiol-inducible CRE-Lox system for cell lineage analysis, three level-1 vectors were assembled into a pAGM4723 level-2 vector. For the first level-1 vector, the chimeric transactivator XVE, the 3′UTR regulatory sequence, and its binding sequence OLexA were PCR amplified from a pER8 vector and cloned into pICH41308, pICH41276 and pICH41295 level-0 vectors, respectively (Zuo et al., 2000). Level-0 vectors containing the *AtCo2* promoter, XVE coding region and XVE 3′UTR region were combined into a pICH47732 level-1 vector. For the second level-1 vector, a SV40 NLS sequence was synthesized (see Table S1) and cloned into a pAGM1276 level-0 vector. A CRE recombinase sequence was PCR amplified from a CRE-GR sequence in pICH41308 and cloned into pICH41308 (Ishida et al., 2016). OLexA, SV40-NLS and CRE recombinase sequences were combined into a pICH47751 level-1 vector. For the third level-1 vector, the *PjACT* promoter sequence in pICH41295 was PCR amplified and cloned into a pAGM1251 level-0 vector. One copy of a mCherry sequence and one copy of a mCherry sequence with a SYP122 sequence with an HSP terminator (1xmCherry-SYP) were cloned using the primers containing LoxP sites into pAGM1311 level –1 vector. These mCherry sequences in level –1 vectors were then assembled with other mCherry sequences into a pAGM1276 level-0 vector to generate floxed 3xmCherry-SYP. The *PjACT* promoter in pAGM1251, LoxP-3xmCherry-SYP-LoxP in pAGM1276, and 3xVenus-SYP in pICH41308 were combined into a pICH47742 level-1 vector to generate PjACT-LoxP-3xmCherry-SYP-LoxP-3xVenus-SYP. All three level-1 vectors were finally assembled into a pAGM4723 level-2 vector to generate an estradiol-inducible CRE/lox construct for cell lineage analysis. For the terminator sequence, a 35S terminator (pICH41414) or a *AtHSP18.2* terminator sequence (Ishida et al., 2016) was used unless specified otherwise.

The PjHB8 promoter sequence (Genbank accession number LC363589) was PCR amplified from *P. japonicum* genomic DNA. A level-2 vector backbone sequence that included the 3xVenus-SYP sequence was PCR amplified. These two fragments were assembled by using the Gibson Assembly method (Gibson et al., 2009), yielding *PjHB8pro::3xVenus-SYP*.

The *PjSCR* promoter sequence (Genbank accession number LC382265) was PCR amplified from *P. japonicum* genomic DNA and inserted into a pENTR vector (Thermo Fisher Scientific). Subsequently, the *PjSCR* promoter sequence was transferred to the binary vector pBGYN using the GATEWAY system (Kubo et al., 2005).

### *P. japonicum* transformation

Transformation was performed as described previously (Ishida et al., 2011). Briefly, 6- or 7-day-old *P. japonicum* seedlings were subjected to 10 s sonication and 5 min vacuum infiltration with water suspension of *Agrobacterium rhizogenes* strain AR1193. This was followed by co-cultivation at 22°C for 2 days in the dark on Gamborg's B5 medium (0.8% agar, 1% sucrose and 450 μM acetosyringone; Sigma). Subsequently, plants were transferred onto Gamborg's B5 medium supplemented with antibiotic (0.8% agar, 1% sucrose and 300 μg/ml cefotaxime; Tokyo Chemical Industry) and incubated at 25°C under long-day conditions.

### Confocal microscopy

Hairy roots were transferred from B5 media to 0.7% INA agar (Funakoshi) with no nutrients to promote their growth along the surface of the solid agar and were incubated for 2 days at 25°C under long-day conditions. Fluorescence-positive plants were placed in specialized small petri dishes with a glass slide on the bottom side (IWAKI, Japan). Hairy roots were sandwiched between thin 0.7% agar (700 μl) on top and the glass slide below and incubated for 1 day at 25°C under long-day conditions. Seven to 10-day-old *A. thaliana* seedlings were carefully placed next to hairy roots under thin agar. Samples were kept at 25°C under long-day conditions and observed continuously with an inverted confocal microscope (Leica SP5 or SP8). For clonal analysis, hairy roots were first incubated on 0.7% INA agar supplemented with 10 μM estradiol in a dish for 24 h for CRE recombinase induction. After Venus fluorescence was confirmed under a confocal microscope, plants were transferred to a new dish with 0.7% agar (INA) without estradiol and were subjected to an infection assay. To excite fluorescence, we used a 405-nm laser for Calcofluor White; a 488-nm laser for GFP, YFP and Venus; a 543-nm laser for Propidium Iodide (PI) and mCherry; and a 561-nm laser for Basic Fuchsin and Direct Red 23. We detected Calcofluor White at 425-475 nm, GFP at 500-540 nm, YFP and Venus at 510-560 nm, mCherry at 570-640, PI at 600-650 nm, Direct Red 23 at 580-615 nm and Basic Fuchsin at 600-650 nm. For expression pattern analysis, we selected hairy roots expressing fluorescent proteins observed commonly among the same constructs, and excluded haustoria that did not complete haustorium development. We considered the hairy roots showing middle levels of expression to be representative and selected these for use in the figures accompanying this report.

## Supplementary Material

Supplementary information
