# Supplementary material for: Induced cell fate transitions at multiple cell layers configure haustorium development in parasitic plants
Source: Development. 2018 Jul 23;145(14):dev164848. doi: 10.1242/dev.164848 (PMC6078332; doi:10.1242/dev.164848)
Supplement: Supplementary information [file develop-145-164848-s1.pdf]

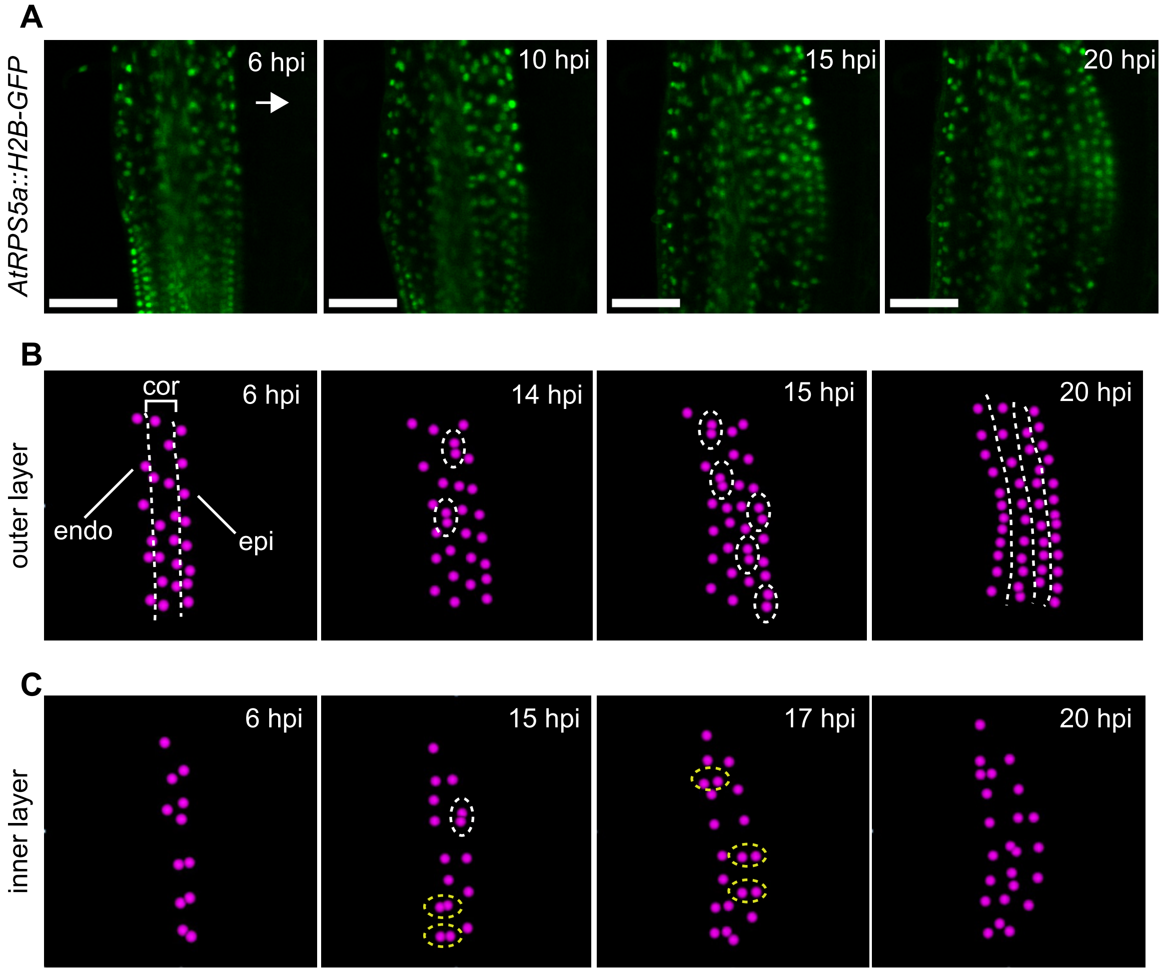

Supplementary figure 1. Nuclei tracking in early developmental stages of haustorium development. (A) Time-lapse observation of cell division during early haustorium development in *P. japonicum* root expressing nuclear localized GFP. Confocal photos were taken at 20 minutes intervals for 14 hours (6 hpi ~ 20 hpi). Selected photos at indicated time points are shown. Arrow indicates the direction of haustorium growth. GFP fluorescence is shown in green. Bar = 100 µm. (B, C) Results of nuclei tracking using the Trackmate software based on sequential confocal photos shown in (A). Purple circle indicates one nucleus. White dotted circles indicate anticlinal divisions. Yellow dotted circles indicate periclinal divisions. (B) Nuclei in the outer four cell layers are shown as indicated. hpi, hours post infection; epi, epidermis; cor, cortex: endo, endodermis. White dotted lines delineate layers. (C) Nuclei in the inner layers are shown.

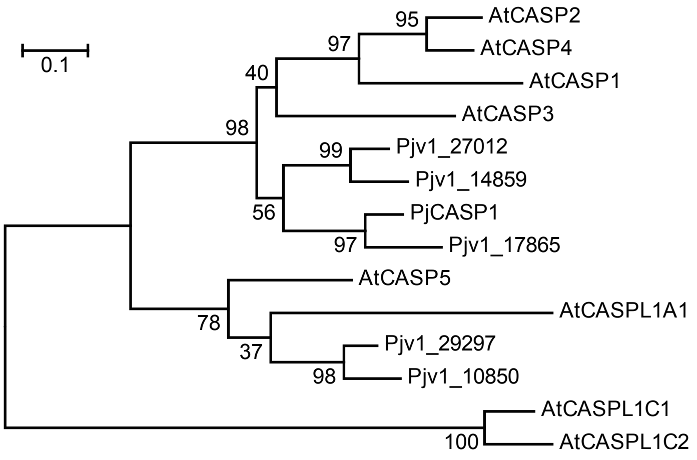

Supplementary figure 2. The phylogenetic tree for *A. thaliana* (At) and *P. japonicum* (Pj) CASP proteins. Amino acid substitutions per site is shown on the scale bar.

Development • Supplementary information

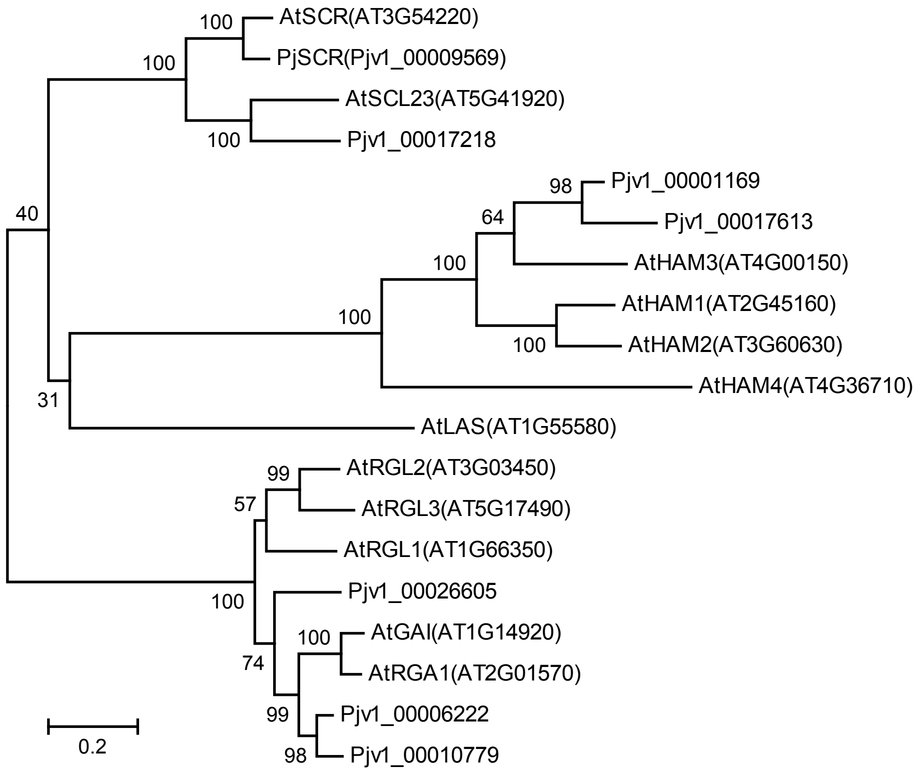

Supplementary figure 3. The phylogenetic tree for *A. thaliana* (At) and *P. japonicum* (Pj) GRAS type transcription factors in subclades SCARECROW(SCR), HAIRY MERISTEM (HAM), and DELLA. Amino acid substitutions per site are shown on the scale bar.

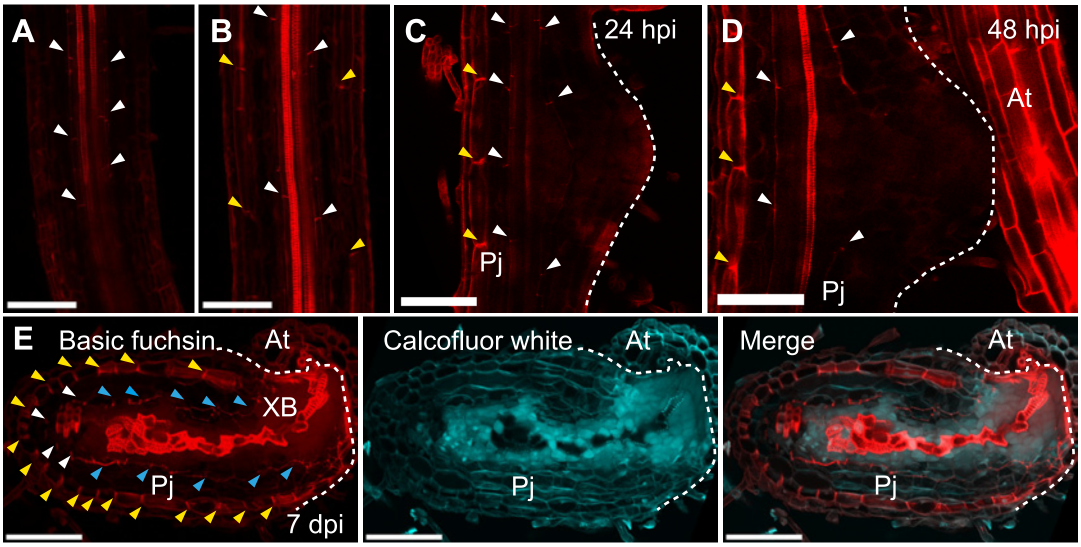

Supplementary figure 4. Casparian strip formation during hasutorium development. (A) The onset of Casparian strip formation in endodermis in *P. japonicum* root. (B) The onset of Casparian strip formation in exodermis in *P. japonicum* root. (C) and (D) Basic fuchsin staining of haustoria at the indicated time points. (E) Basic fuchsin and calcofluor white staning of the hand-sectioned haustorium at 7 days post infection. Basic fuchsin fluorescence is in red. Calcofluor white fluorescent is in cyan. White arrowheads point Casparian strips in endodermis. Yellow arrowheads point Casparian strips in exodermis. Cyan arrowheads point lignin depositions surrounding xylem bridge. White broken lines indicate outlines of haustoria. Maximum projection images from 2 ~ 4 confocal photos are shown. Eight to ten haustoria were observed in each time points. hpi, hours post infction; Pj, *P. japonicum* root; At, *A. thaliana* root; XB, xylem bridge. Bar = 100 µm.

Development • Supplementary information

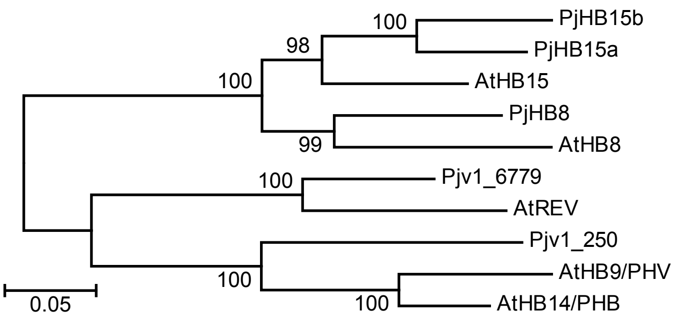

Supplementary figure 5. The phylogenetic tree for *A. thaliana* (At) and *P. japonicum* (Pj) HD-ZIP III transcription factors. Scale bar represents amino acid substitutions per site.

Development • Supplementary information

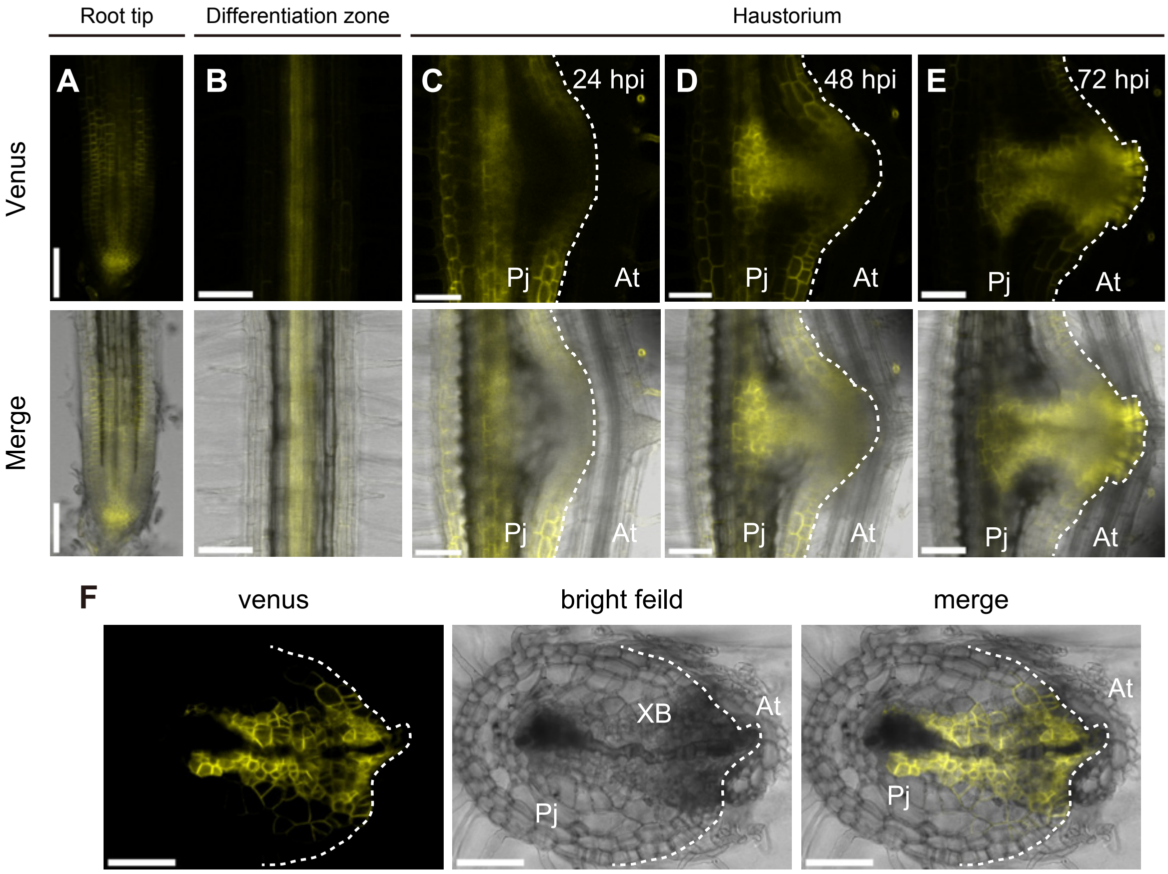

Supplementary figure 6. The expression pattern of *PjHB8::3xVenus-SYP*. The expression patterns in root tip and maturation zone and during haustorium formation are shown as indicated (A)- (E) (n = 4). (F) The hasutorium shown in (E) was hand-sectioned at the middle of the haustorium and examined using a confocal microscope.White line marks the outline of haustorium. Venus fluorescence is in yellow. Bright field images and Venus fluorescent images are merged. Three out of three hairy roots showed the same expression pattern. hpi,hours post infection; Pj, *P. japonicum* root; At, *A. thaliana* root; XB, xylem bridge. Bars = 100 μm.

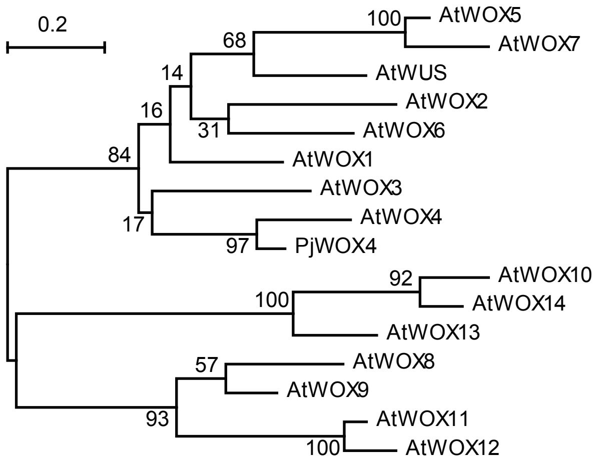

Supplementary figure 7. The phylogenetic tree for *A. thaliana* (At) WOX gene family and *P. japonicum* (Pj) WOX4 .
Scale bar represents amino acid substitutions per site.

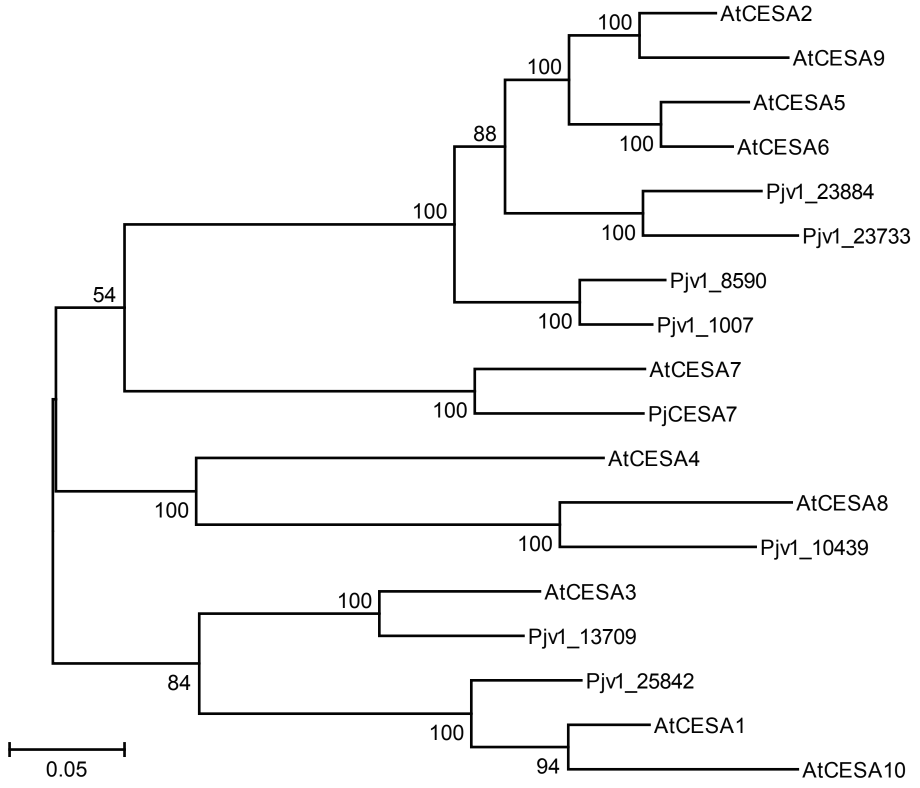

Supplementary figure 8. The phylogenetic tree for *A. thaliana* (At) and *P. japonicum* (Pj) CESA gene family. Scale bar represents amino acid substitutions per site.

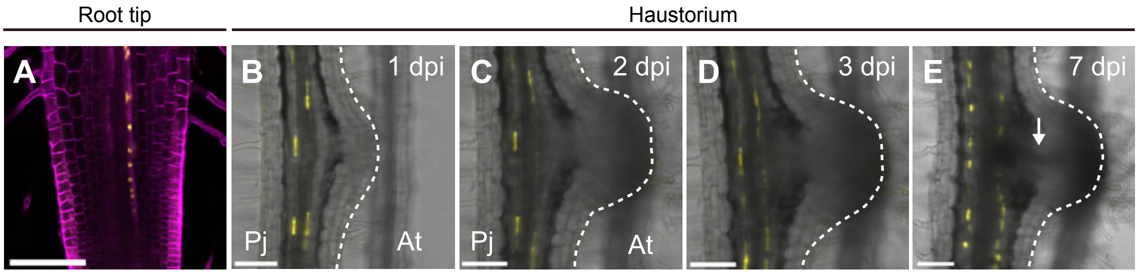

Supplementary figure 9. The expression pattern of *AtAPL::3xVenus-NLS* in *P. japonicum*. (A)-(E) The expression patterns in root tip and during haustorium formation are shown as indicated. White line marks the outline of haustorium. Venus fluorescence is in yellow. Direct red 23 staining is in magenta. A Venus fluorescent images and a direct red 23 fluorescent image are merged in (A) (n = 3). Bright field images and Venus fluorescent images are merged in (B)-(E). The same haustorium is shown in (B)-(E). Five out of seven hairy roots showed the same expression pattern. dpi, days post infection; Pj, *P. japonicum* root; At, *A. thaliana* root. Bars = 100 μm.

Supplemental Table 1

name	sequence	purpose
PjHB15apro-f1	TTGGTCTCAACATGGAGCCGATTGCTTGTTTTGATG	promoter
PjHB15apro-r1	TTGGTCTCAACAAAAATAGAAGGCATGGGGGA	
PjHB15apro-f2	TTGGTCTCAACATATTTTCTCTTTCTCTGCTCATTT	
PjHB15apro-r2	TTGGTCTCAACAACATTCTCAGTCCTTAATTA	
PjHB15bpro-f1	TTGGTCTCAACATGGAGAGGTTGAGTTAGCAGA	promoter
PjHB15bpro-r1	TTGGTCTCAACAACGCAGAAAATGTACCCTAAA	
PjHB15bpro-f2	TTGGTCTCAACATTGCGACATACAAAAAGAAAAGC	
PjHB15bpro-r2	TTGGTCTCAACAACATTCTCTCACTAATTTTTTTTTTCAGC	
PjWOX4-ggf1	TTGGTCTCAACATGGAGTTCACCGGTCCGGTTTTTAAA	promoter
PjWOX4-ggr1	TTGGTCTCAAGCCACTTTTACCACCAAATCT	
PjWOX4-ggf2-2	TTGGTCTCAGGCTCTCATATTCTTAAAAAATGAGA	
PjWOX4-ggr2-2	TTGGTCTCAACAAGTGCTCTCATTAACAGCTGAATG	
PjWOX4-ggf3	TTGGTCTCAACATGCACACACCACATATCCGTATC	
PjWOX4-ggr3	TTGGTCTCAGTAGTAATTAGATGGTGGGGGG	
PjWOX4-ggf4	TTGGTCTCACTACAGACCACTTTCCCATTTC	
PjWOX4-ggr4	TTGGTCTCAACAACATTTTTATGATGGGGGCAAAAT	
PjCASP1pro-ggf1	TTGGTCTCAACATGGAGACGGCGCCAAAATGATG	promoter
PjCASP1pro-ggr1	TTGGTCTCAACAACAATACAATAGAGCAGCCAGT	
PjCASP1pro-ggf2	TTGGTCTCAACATATTGTACGTCGTGATCCTTG	
PjCASP1pro-ggr2	TTGGTCTCAACAACATTCTTCTTTGTTTCTTTTGGTTA	
Venus-NTf1	TTGAAGACAAAATGGTGAGCAAGGGCGA	coding region
Venus-NTr1	TTGAAGACAAACCTTTGTACAGCTCGTCCATGCC	
PjCASP1-ggf3	TTGAAGACAAAGGTATGGAGAAAAGTGAATCAACC	coding region
PjCASP1-ggr3	TTGAAGACAAAAGCTTAATGTTTTTTTAGAGCTATAGCC	
PjCASP1-ggf4	TTGAAGACAAGCTTTCGAACAAAAAAAAAAAGATATATTA	3'UTR region
PjCASP1-ggr4	TTGAAGACAATGACGGGCTTTTCGAG	
PjCASP1-ggf5	TTGAAGACAAGTCATCCAGCCCGGAAA	
PjCASP1-ggr5	TTGAAGACAAAGCGGCCAAAATCGGGAA	
AtCO2-ggf1	TTGAAGACAAGGAGAGTATTGGGCCTTTTG	promoter
AtCO2-ggr1	TTGAAGACAACATTATCGTTATTAACTAGGGTTC	
PjCESA7-ggf1	TTGAAGACAAGGAGTTGACCTGTGTGCCGGA	promoter
PjCESA7-ggr1	TTGAAGACAACATTGGTGGTGGTGGTGTCGAG	
PjACT-ggf1	TTGGTCTCAACATGGAGCTCGCTGGTTTCTTTTAA	promoter
PjACT-ggr1	TTGGTCTCAACAAAGATTTTGAATGGCAATGAG	
PjACT-ggf2	TTGGTCTCAACATATCTAAAATCACTCCCAAAT	
PjACT-ggr2	TTGGTCTCAACAACATTCTCTTATTCTGAGGAACAAA	
AtSYP122-ggf1	TTGGTCTCAACATATCCATGAACGATCTTCTCTCCG	coding region
AtSYP122-ggr1	TTGGTCTCATCCTCGTTTACAACCTCCACG	
AtSYP122-ggf2	TTGGTCTCAAGGACCTTAAAGAGCTAGATCG	
AtSYP122-ggr2	TTGGTCTCAACAAAAGCTTAGCGTAGTAGCCGCC	
LoxmC-f	TTGGTCTCAACATCCATAACTTCGTATAGCATACATTATACGAAGTTATATGGTGAGCAAGGGCG	loxP site
LoxmC-r	TTGGTCTCAACAACTTGTACAGCTCGTCCA	
LoxSYPter-f	TTGGTCTCAACATATCCATGAACGATCTTCTC	loxP site
LoxSYPter-r	TTGGTCTCAACAACATTATAACTTCGTATAATGTATGCTATACGAAGTTATCGCCTCGTTTTACTCTAT	
PjACT-ggff	TTGAAGACAAGGAGCTCGCTGGTTTC	promoter
PjACT-ggfr	TTGAAGACAAATGGCTCTTATTCTGAGGAACAAAAA	
PjHB8-GAf	GTGCATCCTCGTAAGGAGAAAAACCCAAAAAAAGACA	promoter
PjHB8-GAr	CCTTGCTCACCATTCTCACTTCAACAAAATCAAAAGACA	
Lv2SYP-GAf1	GAGAATGGTGAGCAAGGG	vector backbone
Lv2SYP-GAr1	CCTTACGAGGATGCACA	
PjSCRpro-f	CACCATTTGGTGCCTTTCATGGGGTT	promoter
PjSCRpro-r	CTTGGCGGTGCTGCTGCTTT	
AtAPLpro-f1	TTGGTCTCAACATGGAGTGGGGAAAAGATAAGAAG	promoter
AtAPLpro-r1	TTGGTCTCAACAACCCCTAAATTCAATTGCATGC	
AtAPLpro-f2	TTGGTCTCAACATGGGGTTAATCTAAATCGAAGC	
AtAPLpro-r2	TTGGTCTCAACAACATTCTCTCTCTCTCTCTCTCT	

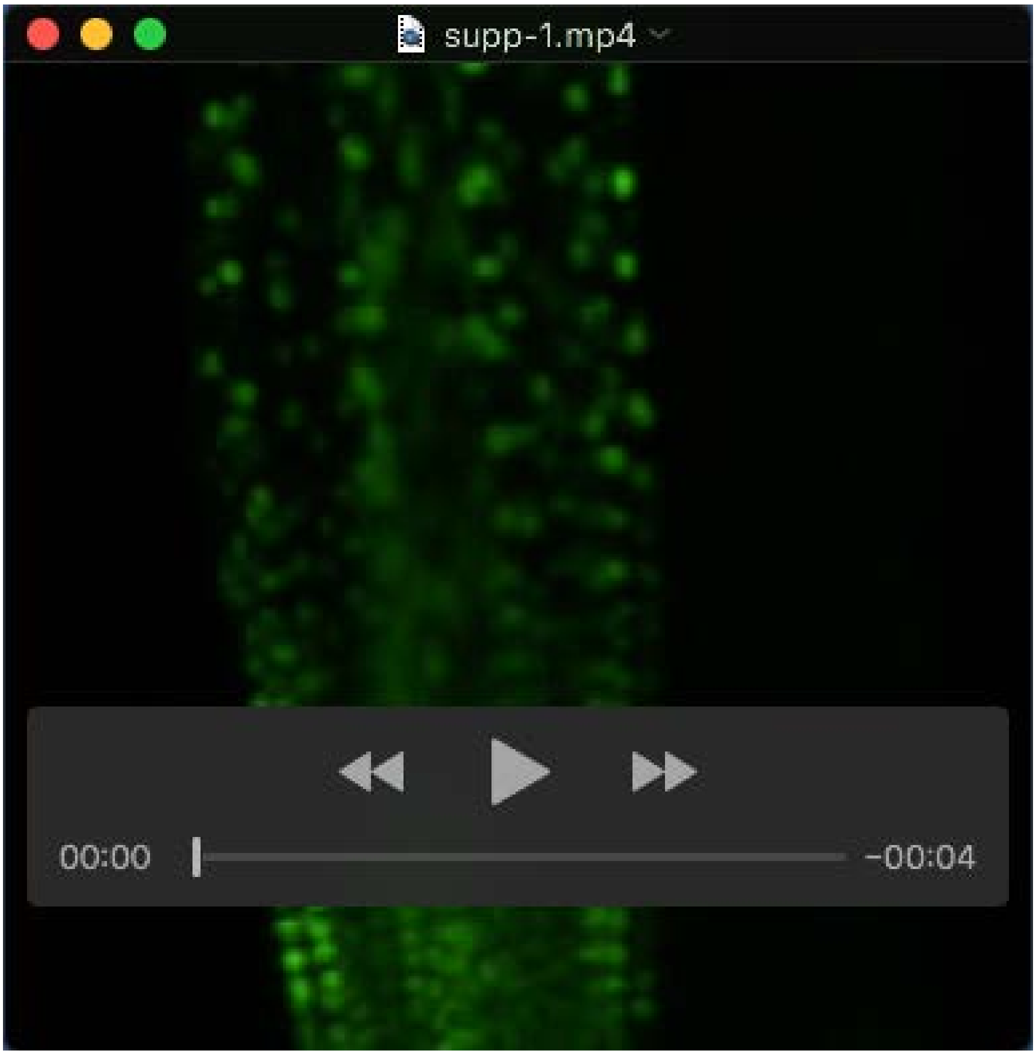

Movie 1. Time-lapse observation of nuclear behavior during early haustorium development. *P. japonicum* root expressing nuclear localized GFP was used to track cell division. Confocal photos were taken at 20 minutes intervals for 14 hours (6 hpi ~ 20 hpi). GFP fluorescent is shown in green.

Development • Supplementary information

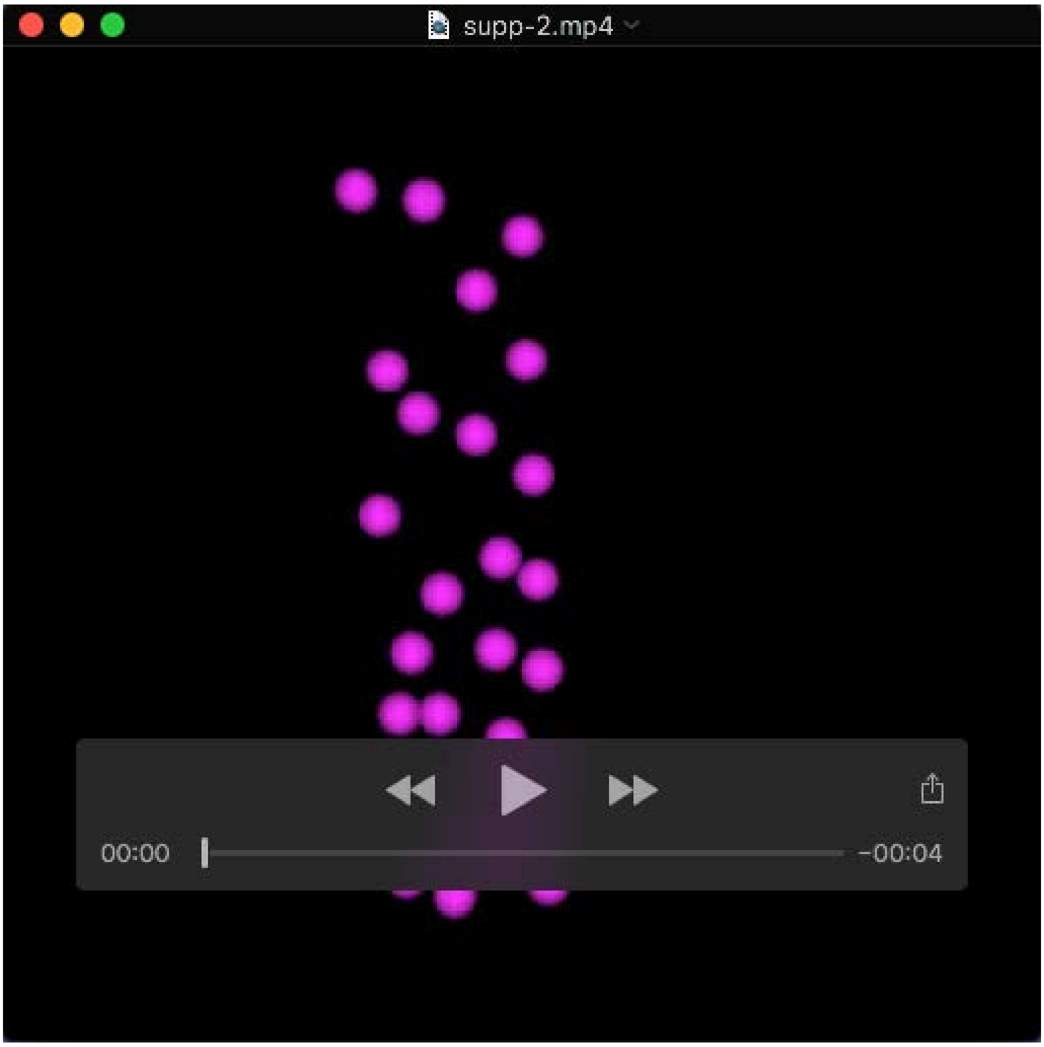

Movie 2. Nuclei tracking in outer cell layers during early haustorium development. The result of nuclei tracking using the Trackmate software based on the time-lapse confocal observation shown in movie 1. Outer cell layers, including epidermis (single layer), cortex (two layers), and endodermis (single layer), are shown from right. Purple circle indicates one nucleus.

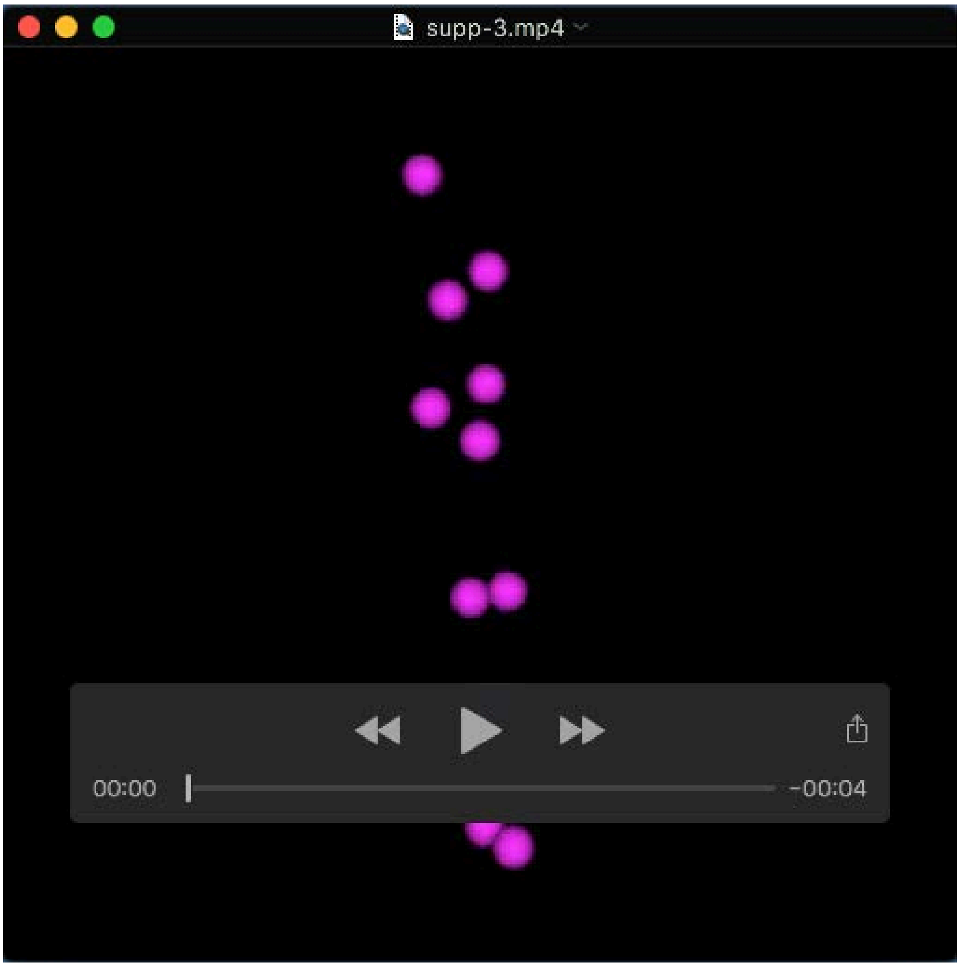

Movie 3. Nuclei tracking in inner cell layers during early haustorium development. The result of nuclei tracking using the Trackmate software based on the time-lapse confocal observation shown in movie 1. Inner cell layers, including pericycle and vascular cell, are shown. Purple circle indicates one nucleus.
